# Sexual behaviours and contraceptive use among undergraduates in a Nigerian University

**DOI:** 10.4314/gmj.v58i2.5

**Published:** 2024-06

**Authors:** Oluwabusola A Akinbajo, Olusoji J Daniel, Adesola O Adekoya, Olufunmilola O Abolurin, Akinkunmi E Akinbajo, Abiola O Adekoya

**Affiliations:** 1 Department of Health Services, Tai Solarin University of Education, Ogun State, Nigeria; 2 Department of Community Medicine and Primary Care, Obafemi Awolowo College of Health Sciences, Olabisi Onabanjo University, Ogun State, Nigeria; 3 Department of Paediatrics, School of Clinical Sciences, Babcock University, Ogun State, Nigeria; 4 Department of Sexual and Reproductive Health, United Nations Population Fund, Nigeria; 5 Department of Radiology, Obafemi Awolowo College of Health Sciences, Olabisi Onabanjo University, Ogun State, Nigeria

**Keywords:** Sexual behaviours, Contraceptives, Undergraduates, Nigeria

## Abstract

**Objectives:**

The study was conducted to describe the sexual behaviours and contraceptive use, as well as factors associated with being engaged in sex among Nigerian undergraduates.

**Design:**

A descriptive cross-sectional study.

**Setting:**

The study was conducted in a university of education in Ogun State, Nigeria.

**Participants:**

Four hundred undergraduates were selected sequentially through a stratified sampling method.

**Main outcome measures:**

Being sexually active, multiple sexual partners, and contraceptive use.

**Results:**

Participants' ages ranged from 16 to 24 years. Nearly two-thirds (65.5%) were females. About a quarter of them (24.5%) were using psychoactive substance(s). More than two-fifths (44.5%) of them had engaged in sexual intercourse, of which 36% had a history of multiple sexual partners. The mean age at sexual debut was 18.7 ± 2.7 years, significantly lower among males than females (p <0.001). About half (218; 54.5%) of the students were cognisant of contraceptives, and 39.3% of sexually active participants had used contraceptives at some time, with condoms being the most commonly used. Male sex (p = 0.042), older age (p <0.001), higher monthly allowance (p = 0.025), and substance abuse (p <0.001) were factors that made undergraduates more likely to have engaged in sex.

**Conclusion:**

Engagement in sexual activity and unsafe sex were common practices among the Nigerian undergraduates studied. It is recommended that deliberate efforts be made to increase contraceptive awareness and usage.

**Funding:**

None

## Introduction

Sexual health is an important component of the overall health of an individual.^1^ Sexual health includes issues of sexual development, sexual orientation, sexual behaviours, pregnancy, contraception as well as sexually transmitted diseases. Promotion of sexual health is highly beneficial as it greatly enhances general well-being and functioning, thereby preventing several other medical conditions, including physical and psychological ill-health.^1^ Sexual health may be influenced by various factors such as personal values and beliefs, culture, religion and spirituality, socioeconomic factors, societal influences, and physical health.^1^

The term ‘youth’ refers to the period between childhood and adulthood and is defined by the United Nations as those persons aged between 15 and 24 years.^2^ Youths constitute about 16 per cent of the global population and are generally recognised as agents of change based on their energy and potential.^3^

As youths become independent and more exposed, striking changes may occur in their behaviour, reasoning, and decision-making. In addition, physical development associated with puberty becomes more pronounced, and sexual maturation is heightened.^4^

During this period, some youths get exposed to illicit drugs, alcohol, and cigarette smoking, and many experience initiation of sexual activity.^5^

Initiation of sexual activity may vary by region, country, and sex.^6^ Globally, young people are reaching puberty earlier than in the past and becoming sexually active at younger ages.^7^ Unsafe sexual practices, which can lead to negative consequences, are commonly practised by youths.^5^ These include unprotected sex, multiple sexual partners, and having sex while under the influence of alcohol or drugs. Hence, sexually transmitted diseases (STDs) and pregnancy complications, including abortion sequelae, constitute important causes of morbidity and mortality among youths worldwide.^8^,^9^

Based on the fact that a sizeable number of young people experience the adverse health consequences of early, unprotected sexual activity, it has been advocated that education on contraception and, more importantly, access to contraception should be greatly improved among adolescents and youths.^6^,^7^ In fact, knowledge and use of contraceptives are considered important indicators of the sexual health of adolescents and youths.^6^,^7^ High levels of contraceptive awareness have been reported in Nigeria, but the level of utilisation of these contraceptives has been disproportionately low.^10^–^12^ Among adolescents and youths, the male condom has been featured as the most popular contraceptive.^10^,^13^,^14^

Understanding the factors that influence sexual behaviours among youths is essential in reducing the negative consequences of unsafe sexual practices and promoting positive sexual health among them. Previous studies have shown that as many as half to two-thirds of Nigerian undergraduates might have experienced sexual intercourse, with a similar proportion of these sexually active students being involved in risky sexual behaviours such as unprotected sex, multiple sexual partners, and having sex under the influence of alcohol. Most sexual debuts occurred between the ages of 16-20 years.^10^,^13^,^15^–^17^ Male sex and family background were found to influence sexual behaviours among the undergraduates significantly.^13^,^15^,^17^ The sexual behaviours of Nigerian youths, particularly those in tertiary institutions, are yet to be fully explored. This study was therefore carried out to determine the pattern of sexual behaviours and contraceptive use among Nigerian undergraduates.

## Methods

The study was conducted at the University of Education, Ijagun, Ogun State, Nigeria, between January and March 2019. The university was chosen based on accessibility and convenience.

The university has five colleges, each comprising four to six departments. Each of the courses offered runs for four years. The participants were undergraduates who had spent at least one year on campus. The study employed a descriptive cross-sectional design in which participants were selected sequentially through a stratified sampling method based on the faculty/department of study, level of study, and class size. A minimum sample size of 358 participants was obtained using Fisher's formula^18^ for sample size calculation with absolute sampling error set at 5%, normal standard deviate taken as 1.96, and proportion taken as 0.63 according to a previous study^15^, which revealed that 63% of university undergraduates were sexually active. Considering a non-response rate of 10%, a sample size of 400 students was employed for the study. Exclusion criteria included first-year (100 level) students due to their short duration on campus, students aged 25 years and above, and married students.

Data was collected using a pre-tested, structured, self-administered questionnaire that featured information on socio-demographic characteristics, substance use, sexual behaviours (including sexual activity), as well as knowledge and use of contraception. In this study, sexual activity was defined as ‘ever engaged in sexual intercourse’. Data were entered into the Statistical Package for the Social Sciences (SPSS) version 21.0 for analysis. Data were presented using descriptive statistics; mean and standard deviation were used to summarise continuous variables, while frequencies and percentages were calculated for categorical variables. The characteristics associated with engaging in sex were determined using the chi-square test. P-values less than 0.05 were considered statistically significant.

Ethical approval for the study was obtained from the Olabisi Onabanjo University Teaching Hospital Health Research Ethics Committee (OOUTH/HREC/219/2018AP), and permission was also obtained from the authorities of the University of Education to conduct the study. Written informed consent was obtained from each study participant after they were assured of privacy and confidentiality. All information regarding the respondents was treated with the utmost confidentiality.

## Results

### Socio-demographic characteristics

A total of 420 questionnaires were distributed. Of these, 400 were appropriately filled, while 20 were either incompletely filled or not filled at all, and were therefore excluded from the analysis. The socio-demographic characteristics of the 400 study participants are shown in Table 1. They were between 16 and 24 years, with a mean age of 21.3 ± 1.9 years.

**Table 1:** Socio-demographic characteristics of the respondents

Characteristic	n (%)
**Age group**	
16-19 years (Adolescents)	69 (17.3)
20-24 years (Young adults)	331 (82.7)

**Sex**	
Male	138 (34.5)
Female	262 (65.5)

**Religion**	
Christianity	276 (69.0)
Islam	120 (30.0)
Traditional	4(1.0)

**Monthly allowance**	
≤₦10,000	302 (75.5)
₦11,000 - ₦20,000	54 (13.5)
>₦20,000	44 (11.0)

**Level (Year) of study**	
200 level	138 (34.5)
300 level	119 (29.8)
400 level	143 (35.7)

**College of study**	
Science and Information Technology	118 (29.5)
Specialised and Professional Education	73 (18.2)
Humanities	51 (12.8)
Social and Management Sciences	102 (25.5)
Vocational and Technology Education	56 (14.0)

Sixty-nine (17.3%) of them were in their teenage years, about one-third (34.5%) were males, and more than two-thirds (69.0%) were Christians. The majority (75.5%) received a monthly allowance of less than ₦10,000 (28 USD), and more than one-third (35.8%) were in their final year of study (400 level). The Colleges of Science and Information Technology and Social and Management Sciences had the most participants.

### Sexual practices

Table 2 shows the sexual behaviours of the respondents. More than two-fifths (178; 44.5%) of the respondents had engaged in sexual intercourse. The circumstances leading to sexual debut among them were willingness (62.9%), persuasion (30.3%), or an act of force (6.8%), while the partner at sexual debut was the boyfriend/girlfriend in most (87.1%) cases. Age at sexual debut ranged from 7 to 24 years with a mean of 18.7 ± 2.7 years. History of multiple sexual partners was present in 64 (36.0%), while more than a quarter (28.7%) had sex recently (within one week before data collection) as shown in Table 2. Sixtyseven (16.8%) admitted that they had practised oral sex, while 19 (4.8%) agreed to anal sex; the others practised only vaginal sex.

**Table 2:** Sexual behaviours among the respondents

Sexual Characteristic	n (%)
**Ever engaged in sex (n = 400)**	
Yes	178 (44.5)
No	222 (55.5)

Number of sexual partners in the preceding one year (n = 178)*	
None	23 (12.9)
1	91 (51.1)
2	42 (23.6)
≥3	22 (12.4)

Last sexual experience (n = 178)*	
Within the previous week	51 (28.7)
1-4 weeks	31 (17.4)
1-3 months	17 (9.6)
3-6 months	22 (12.4)
>6 months	30 (16.8)
Not reported	27 (15.2)

Circumstances leading to sexual debut (n = 178)*	
Willingness	112 (62.9)
Persuasion	54 (30.3)
Act of force	12 (6.8)

Partner at sexual debut (n = 178)*	
Boyfriend/girlfriend	155 (87.1)
Playmate/classmate	19 (10.7)
Older neighbour/acquaintance	4 (2.2%)

Practice of non-vaginal sex (n = 178)*	
Oral sex	67 (16.8%)
Anal sex	19 (4.8%)

* Applies to only the sexually active respondents.

Table 3 compares sexual behaviours between males and females. Sexual activity was significantly more prevalent among males than females (p = 0.042). Age at sexual debut was also significantly lower among the males (p <0.001), and a higher proportion of males had multiple sexual partners (p <0.001). There were, however, no significant differences in the proportions of those who practised oral/anal sex between the male and the female students (p = 0.690).

**Table 3:** Comparison of sexual behaviours between male and female students

	Sex	
	Male n (%)	Female n (%)
**Ever engaged in sexual intercourse**		
**Yes**	**71 (51.4)**	**107 (40.8)**
**No**	**67 (48.6)**	**155 (59.2)**
	**χ^2^ = 4.12, p = 0.042**	
**Age at sexual debut**	**17.4 ± 3.2 years**	**19.4 ± 2.1 years**
	**t = -4.66; p <0.001**	
**Circumstances leading to sexual debut**		
**Willingly**	**48 (67.6)**	**64 (59.8)**
**Persuasion**	**19 (26.8)**	**35 (32.7)**
**Act of force**	**4 (5.6)**	**8 (7.5)**
	**χ^2^ = 1.13, p = 0.570**	
**Multiple sexual partners**		
**Yes**	**37 (26.8)**	**27 (10.3)**
**No**	**101 (73.2)**	**235 (89.7)**
	**χ^2^ = 18.32, p <0.001**	
**Practices oral/anal sex**		
**Yes**	**27 (19.6)**	**47 (17.9)**
**No**	**111 (80.4)**	**215 (82.1)**
	**χ^2^ = 0.16, p = 0.690**	

### Sex-related outcomes

A previous history of abnormal vagina or penile discharge, suggestive of STI, was positive in 58 (14.5%). Eighteen (6.9%) of the females (n = 262) had been pregnant before, while 8 (5.8%) of the males (n =138) had impregnated someone in the past. Outcomes of pregnancy among the 18 females included child delivery in 6 and termination of pregnancy in 12 of them.

### Contraceptive knowledge and use

More than half of the 400 students (218; 54.5%) were cognisant of contraceptives. The source of information on contraceptives was the media in 93 (42.7%), friends in 59 (27.1%), health facilities in 35 (16.1%), and the parents or siblings in 28 (12.8%) and 3 (1.4%), respectively. Of the 178 students who had engaged in sex, 70 (39.3%) had used contraceptives at some time, of which 41 were currently using contraceptives. Condoms (54/70; 77.1%) were the most common contraceptive used, followed by oral contraceptive pills (25/70; 35.7%); implants (7; 10.0%) and intrauterine devices (5; 7.1%) were less common. Sixteen (22.9%) of them had used emergency contraception (morning-after pills) before.

### Substance use

Ninety-eight (24.5%) of the respondents reported using a psychoactive substance(s), with the use of multiple substances being common practice.

**Figure 1:**
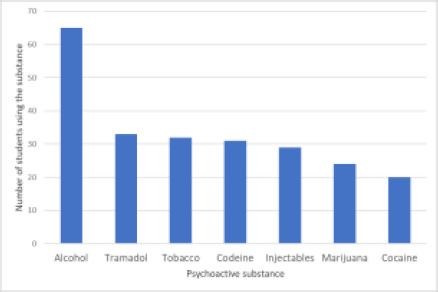
Psychoactive substances being used by the respondents *Multiple responses allowed

The most commonly used substance was alcohol (n = 65; 16.2%), followed by tramadol, codeine, and tobacco as shown in Figure 1. Other substances being used were marijuana, cocaine, and injectables.

### Factors associated with sexual activity

Table 4 shows the factors associated with sexual activity among the respondents.

**Table 4:** Factors associated with sexual activity among the respondents

	Ever engaged in sexual intercourse	
	Yes n (%)	No n (%)
**Age-group**		
16-19 years	14 (20.3)	55 (79.7)
≥20 years	164 (49.5)	167 (50.5)
	χ^2^ = 19.79, p <0.001	
**Sex**		
Male	71 (51.4)	67 (48.6)
Female	107 (40.8)	155 (59.2)
	χ^2^ = 4.12, p = 0.042	
**Religion**		
Christianity	124 (44.9)	152 (55.1)
Islam	51 (42.5)	69 (57.5)
Traditional	3 (75.0)	1 (25.0)
	χ^2^ = 1.75, p = 0.423*	
**Monthly Allowance**		
≤₦10,000	128 (42.4)	174 (57.6)
₦11,000 - ₦20,000	22 (40.7)	32 (59.3)
>₦20,000	28 (63.6)	16 (36.4)
	χ^2^ = 7.38, p = 0.025	
**Substance use (any substance)**		
Yes	81 (82.7)	17 (17.3)
No	97 (32.1)	205 (67.9)
	χ^2^ = 76.50, p <0.001	

* *Likelihood ratio applied*.

A significantly higher proportion of the males had engaged in sex compared with females (p = 0.042), and older students were more likely to have engaged in sex than the younger ones (p <0.001). Furthermore, students who received greater than ₦20,000 (56 USD) as monthly allowance reported more sexual activity than those who received less (p = 0.025). Substance abuse was also significantly associated with sexual activity (p <0.001), whereas religion did not influence sexual activity (p = 0.423).

## Discussion

Undergraduate youths usually experience a great degree of freedom of activities because parental monitoring and control are usually minimal on campus. Sexual exploration is, therefore, a common phenomenon and is often triggered by curiosity and peer pressure.^19^ Nearly half of the students in the present study had engaged in sex, and having multiple sexual partners was a common practice among them. The proportion of students who had engaged in sex was much lower than in many previous reports from Africa and other continents.^13^,^15^,^16^,^20^–^22^

However, some other studies have reported lower proportions.^12^,^19^ The Inclusion of married students in some of the previous studies may partly account for the higher rates of sexual activities observed. Furthermore, the varying demands and rigorousness of different courses of study may influence the level of involvement in sexual activities among diverse student populations. Differences in the rules and regulations guiding sexual activities on campus, as well as accommodation settings and levels of monitoring across the institutions, may also contribute to the variations observed.

The mean age at sexual debut in our study was about 18 years, which is similar to the findings in previous Nigerian and African studies,^17^,^22^–^24^, but much higher than in America.^25^ The lower age we observed among the males compared to females also agrees with previous reports.^13^,^26^ However, some other studies found sexual initiation to be earlier among females.^27^,^28^ An interplay of several factors which have been found to influence the onset of sexual activity may account for these differences. Such factors include family relationships, friends' values and actions, socio-cultural factors, substance use, watching pornography, and the influence of social media.^17^,^26^–^29^ In agreement with our findings, previous studies among undergraduates have also shown that the partner at sexual debut is commonly the boyfriend or girlfriend, and most sexual debuts are voluntary. Forced sexual debut, considered as rape, was similarly reported.^12^,^15^,^20^,^23^

More of the males in our study were sexually active and had multiple sexual partners compared to the females. This is not surprising as males typically have a stronger sex drive than females. Our study also revealed that nearly one-fifth of the students practised oral and/or anal sex, as documented in Southeast Nigeria in earlier studies.^16^,^23^ Contrastingly, only vaginal sex was practised by undergraduates in a previous study carried out in Northwest Nigeria.^12^ The reasons for the differences may need further exploration, though religious and socio-cultural inclinations may be contributory.

Risky sexual practices, of which multiple sexual partners were found to be the most common in this study, have also been previously noted to be popular among undergraduates.^13^,^16^,^17^,^19^ These practices greatly increase the risk of sexually transmitted infections, features of which were present in more than a tenth of the study participants. Furthermore, risky behaviours commonly result in unintended pregnancies, which are frequently aborted, as demonstrated in the present study in which two-thirds of the ladies who were pregnant terminated the pregnancy. Similarly, a previous study reported a high rate of pregnancy termination among Nigerian female undergraduates.^16^ This is worrisome because post-abortion complications have been reported to be among the leading causes of death among young girls, particularly in Africa.^9^

The knowledge of contraception was relatively low among the youth studied, as just over half were cognisant of it. Knowledge about contraception was found to be higher in previous studies conducted among this age group, with 70-100% of the respondents being aware of contraception.^12^,^14^,^30^,^31^ The reason for the relatively low knowledge found in this study is not clear; however, the hesitance to reveal details of sexual practices due to the biased societal perception may be contributory. Moreover, familiarity with the term contraception does not necessarily equate to being knowledgeable about what it entails, as demonstrated in a previous study where all respondents responded positively to being aware of the term. Still, many of them did not know what it is all about.^30^ The media and friends, being the main sources of information about contraceptives among youth, agreed with previous reports.^12^,^30^,^31^ This implies that healthcare providers have a lot to do in providing information on contraception to the youth.

About one-third of the sexually active students had used contraception, condoms being the most common. Likewise, a previous study found a similar proportion (34.2%) of youth to have used some form of contraception during their last sexual experience.^14^ A relatively higher proportion of sexually active female undergraduates reported using contraceptives in Southern Nigeria.^31^ In contrast, a much lower prevalence of contraceptive utilisation of 15.6% was found among sexually active students in Northern Nigeria.^12^ As in our study, condoms were found to be the most popular contraceptive type among youths in previous studies.^10^,^13^,^14^ Condoms are commonly used due to their accessibility, affordability, and ease of use.

The pattern of substance use in this study featured alcohol as the most common, followed by tramadol and tobacco, as observed in previous studies.^32^,^33^ The common use of alcohol is likely related to its wide availability and unregulated accessibility. Substance use is known to be a major influencing factor in sexual activity and risky sexual behaviours among youths.^34^,^35^ It is possible that the substances are taken in anticipation of a wild sexual experience. This may increase the likelihood of unprotected sex, thereby heightening the chances of STIs and unwanted pregnancies.^36^

Males were more likely to have engaged in sex than females, and age also influenced sexual activity, with older students being more involved than the younger ones. Higher sexual activity among male undergraduate students had been previously documented.^13^,^15^ Students who received higher monthly allowance were more likely to have engaged in sex, compared with those who received less money. Having more money may be more attractive to the opposite sex. In addition, we found that substance use was significantly associated with being sexually active, further corroborating the findings in previous studies which have reported significantly higher rates of sexual activity, including risky sexual behaviours among youths who use psychoactive substances, compared with those who do not.^34^,^35^,^37^

### Limitations

The information obtained in the study was personal and sensitive, and some of the students may have concealed some information.

## Conclusion

In conclusion, sexual intercourse is a common practice among the undergraduates studied, with male sex, older age, and substance use being significantly associated factors. Risky sexual practices were also common, while contraceptive knowledge and use were low. To improve safe sex practices among undergraduates, it is recommended that deliberate efforts be made to increase awareness and usage of contraceptives and discourage the use of illicit substances, particularly among males.
